# Seasonal Timing of Infant Bronchiolitis, Apnea and Sudden Unexplained Infant Death

**DOI:** 10.1371/journal.pone.0158521

**Published:** 2016-07-12

**Authors:** Chantel D. Sloan, Tebeb Gebretsadik, Christian Rosas-Salazar, Pingsheng Wu, Kecia N. Carroll, Edward Mitchel, Larry J. Anderson, Emma K. Larkin, Tina V. Hartert

**Affiliations:** 1 Department of Health Science, Brigham Young University, Provo, Utah, United States of America; 2 Center for Asthma Research, Vanderbilt University School of Medicine, Nashville, Tennessee, United States of America; 3 Department of Biostatistics, Vanderbilt University School of Medicine, Nashville, Tennessee, United States of America; 4 Department of Pediatrics, Vanderbilt University School of Medicine, Nashville, Tennessee, United States of America; 5 Department of Medicine, Vanderbilt University School of Medicine, Nashville, Tennessee, United States of America; 6 Department of Health Policy,Vanderbilt University School of Medicine, Nashville, Tennessee, United States of America; 7 Department of Pediatrics, Pediatric Infectious Diseases, Emory University School of Medicine, Atlanta, Georgia, United States of America; University of Iowa, UNITED STATES

## Abstract

Rates of Sudden Unexplained Infant Death (SUID), bronchiolitis, and central apnea increase in winter in temperate climates. Though associations between these three conditions are suggested, more work is required to establish if there is a causal pathway linking bronchiolitis to SUID through inducing central apnea. Utilizing a large population-based cohort of infants studied over a 20-year period (n = 834,595, from birth years 1989–2009)), we analyzed ecological associations between timing of SUID cases, bronchiolitis, and apnea healthcare visits. Data were analyzed between 2013 and 2015. We used a Cox Proportional Hazards model to analyze possible interactions between maternal smoking and maternal asthma with infant bronchiolitis on time to SUID. SUID and bronchiolitis both occurred more frequently in winter. An increase in bronchiolitis clinical visits occurred within a few days prior to apnea visits. We found a temporal relationship between infant bronchiolitis and apnea. In contrast, no peak in SUID cases was seen during peaks of bronchiolitis. Among those without any bronchiolitis visits, maternal smoking was associated with an increased risk of SUID: Hazard Ratio (HR) of 2.38 (95% CI: 2.11, 2.67, p-value <0.001). Maternal asthma was associated with an increased risk of SUID among infants with at least one bronchiolitis visit: HR of 2.40 (95% CI: 1.04, 5.54, p-value = 0.04). Consistent trends between bronchiolitis, apnea, and SUID were not established due to small numbers of SUID cases. However, interaction analysis revealed potential differential associations of bronchiolitis and SUID by maternal smoking, maternal asthma status.

## Introduction

Sudden Unexplained Infant Death (SUID) is the death of a child less than 1 year old due to a cause that cannot be otherwise defined or detected. Studying biologically plausible causative pathways and risk factors is of the utmost importance to develop further preventive interventions and further reduce risk of SUID [1,2]. Research suggests a link between SUID and central apnea, in which an infant’s regular breathing pattern is halted for several seconds (typically more than 15)[3]. Central apnea may be induced by several different environmental factors, including viral infections. Respiratory syncytial virus (RSV) infects nearly every child by two years of age, and is the most common cause of bronchiolitis among infants [4]. RSV has become an important target in the study of viral induction of central apnea and has been suggested to be a risk factor for SUID [4–8].

Etiologic pathways between RSV and central apnea may be the direct result of viral infection, or may operate through the combination of infection and a second environmental risk factor such as second hand smoke. RSV enters through the upper airway and infects the nasal respiratory epithelium. Infection can then result in the upregulation of chemoreceptors on respiratory sensory neurons and signal neurotrophin release, which can enhance the respiratory inflammatory response [9–11]. Environmental factors, such as nicotine, then bind to and overstimulate chemoreceptors [12,13]. This overstimulation increases sensitivity of the central nervous system to gamma-aminobutyric acid (GABA), adenosine, and other neurotransmitters that can decrease or halt respiration [14,15].

Given the difficulty in addressing a possible link between RSV, apnea, and SUID because of the rarity of these events and the obvious ethical reasons that human experimental studies could never be performed, we undertook an ecological design using a population-based cohort of 20 years of infant births and follow-up. In addition, to investigate plausible effect modifications of infant infection, environmental exposures (maternal smoking exposure) and familial risk factors (maternal asthma) on SUID, we used individual-level data.

## Methods

This study was approved by Vanderbilt University's Institutional Review Board. Consent was not required, as we analyzed medical records with personal identifiers removed. We used a previously described retrospective birth cohort of children enrolled in Tennessee Medicaid (TennCare) linked with administrative and vital records called the Tennessee Asthma and Bronchiolitis Study (TABS) [16,17]. There were N = 836,041, children and of these, we excluded 1,446 infants who had congenital heart disease (0.17%). Eligible children (n = 834,595, from birth years 1989–2009) were followed from birth to one year. Data were analyzed between 2013 and 2015. Maternal asthma was ascertained for infants (n = 329,483) whose mothers met the continuous enrollment criteria of no more than 45 days of non-enrollment. This study investigated all infants, including those that were premature (<37 weeks gestation). Descriptive statistics were calculated as medians with an interquartile range (median [IQR]) or frequencies and proportions by study subgroups (infants with SUID, bronchiolitis, or apnea). We calculated rates that accounted for person time follow-up.

Infant health care visits for bronchiolitis, SUID and apnea were defined using ICD-9 codes as described in the supplemental material [18]. SUID, bronchiolitis and apnea events occurring during birth hospitalization were removed. Bronchiolitis during RSV season among infants <6 months of age was used as a surrogate for RSV infection. For each infant with an apnea healthcare and bronchiolitis healthcare visit, we calculated the number of days between their first bronchiolitis visit and any apnea visits, and the number of days between their first apnea visit and any bronchiolitis visits. We examined trends of any bronchiolitis, apnea, or SUID event by year of study cohort (number for a specific year/denominator of cohort for specific year), as well as the percent of events occurring by calendar month.

### Statistical analysis

Descriptive analyses were done, reporting median and interquartile range [IQR] for continuous variables and frequency and numbers for categorical variables. For comparisons of characteristics between two independent groups (eg.SUID vs. non-SUID) we used Chi-square for categorical variables and Wilcoxon non-parametric test for continuous variables. Exact Poisson confidence intervals were calculated for rates [19]. For temporal relationships, we examined trends of any bronchiolitis, apnea, or SUID event by year of study (number for a specific year/denominator of cohort for specific year). In addition, among subjects with condition(s) of interest (any bronchiolitis [exposure], SUID or any apnea [outcomes]), we examined the percent of events occurring by calendar month for seasonal trends. To examine the timing relationship between bronchiolitis or apnea relative to SUID, we calculated time intervals (in days from date of birth) and present them graphically.

Finally, we used a Cox Proportional Hazards (CPH) model to investigate potential interactions between infant bronchiolitis and maternal smoking with SUID, while accounting for the varying follow-up times inherent in the data (as infants die from SUID at different ages). Bronchiolitis and maternal smoking, and bronchiolitis and maternal asthma cross-products were tested separately.

Covariates included were infant sex, gestational age, birth weight, and year of birth. Stratified analyses by subgroup of infants with no bronchiolitis visits and those who had at least one bronchiolitis visit were also done to assess the relationship of maternal smoking or asthma with SUID events in these groups. Proportional hazard assumptions were checked using the Schoenfeld residuals method. Hazard ratios (HRs) and their 95% confidence intervals (95% CIs) are reported.

## Results

### Population description

Among 834,595 infants, 60% (n = 504,842) were white and 32% (n = 267,794) were African American (Table 1). Median birth weight was 3,232 grams (IQR: 2,863, 3,555), and median gestational age was 274 days (IQR: 267, 281). 8,139 (0.98%) had at least one apnea healthcare visit in their first year of life and 162,607 (19.5%) had a bronchiolitis healthcare visit. Among those who had a bronchiolitis visit, 24.2% (n = 39,435) were a hospital visit. Comparisons in outcome (SUID or no SUID) and exposure (apnea and bronchiolitis) variables for premature and term infants are shown in Table 1 and S1 Table.

**Table 1:** Maternal and Infant Characteristics by Sudden Unexplained Infant Death Status.

Characteristic:		Total Population (N = 834,595)	Infants without SUID (N = 833,400)	Infants with SUID (N = 1,195)
***Exposure Variables***				
	Apnea	8,139^a^	8,129^a^ (1%)	10 (1%)
	Bronchiolitis	162,607^b^	162,548^b^ (20%)	59 (5%)
***Demographic Variables***^***c***^				
**Sex**				
	Male	427179 (51%)	426470 (51%)	709 (59%)
	Female	407407 (49%)	406921 (49%)	486 (41%)
**Race**				
	White	504842 (60%)	504169 (60%)	673 (56%)
	Black	267794 (32%)	267301 (32%)	493 (41%)
	Other	61959 (7%)	61930 (7%)	29 (3%)
**Maternal Age** (Median, [IQR])		22 [19,26]	22 [19,26]	21 [19,25]
**Gestational Age in Days** (Median, [IQR])		274 [267,281]	274 [267,281]	274 [258,281]
**Birth weight** (Median, [IQR])		3232 [2863,3555]	3232 [2863,3556]	2977 [2466,3320]
**Premature birth (< 37 weeks)**		114577 (14%)	114278 (14%)	299 (25%)
**Maternal Smoking**		228011 (27%)	227436 (27%)	575 (48%)
**Maternal Asthma**^**d**^		26924 (8%)	26874 (8%)	50 (8%)

The table presents characteristics for the population of infants born in the Tennessee Medicaid program from 1989–2009 (n = 834,595) by SUID vs. No SUID

^a^Represents those with at least one apnea clinical visit

^b^Represents those with at least one bronchiolitis clinical visit

^c^There were very few infants missing data for the demographic variables (except for maternal asthma, as noted).

^d^The maternal asthma variable had 329,483 infants with available data due to continuous enrollment criteria of no more than 45 days of non-enrollment.

Infants with SUID vs. those without (Table 1) had a lower gestational age distribution (274 days [IQR:258, 281] vs 274 days [267, 281]; higher proportion of maternal smoking (48% vs. 27%) and lower proportion of at least one bronchiolitis visit (5% vs. 20%) (all p<0.001). Infants with SUID and those without had a similar proportion of at least one apnea occurrence (~1%, p value = 0.6) and similar proportion of mothers with asthma (~8%, p value = 0.9). Percentages of exposure variables were similar in premature infants and term infants though they were always higher in the premature group. SUID was higher in the premature group (S1 Table).

### Infants with Apnea

Infants with apnea were more likely to have mothers who smoke compared to the total population (infants with apnea: 37% vs. no apnea: 27%, p <0.001), were more likely to be white compared to the entire group (75% vs. 60%, p <0.001), and be of lower gestational age (Median 263 days [IQR: 232, 274] vs. 274 days [IQR: 267, 281], p <0.001). For the subset of infants with maternal asthma ascertained (329,483), infants with apnea were more likely to have a history of maternal asthma (infants with apnea: 16% vs. no apnea: 8%). Temporal relationships between bronchiolitis and SUID did not change by year of death (data not shown to protect confidentiality).

### Disease Rates

Rates of apnea and SUID events for those with and without a bronchiolitis visit and those with and without an apnea visit are shown in Table 2. Among those infants with no bronchiolitis visits, 12.34/1,000 experienced an apnea event, and 1.70/1,000 died from SUID. For those with at least one bronchiolitis visit, 44.5/1,000 experienced at least one apnea event and 0.36/1,000 died from SUID. Rates of SUID were slightly higher among infants with no apnea visits (1.44/1,000) compared to those with at least one apnea event (1.24/1,000).

**Table 2:** Annualized rates of apnea or SUID by bronchiolitis or apnea visit status.

	No Bronchiolitis	Bronchiolitis	No Apnea	Apnea
Rate* of Apnea	12.34 (12.07, 12.61)	44.5 (43.45,45.51)	NA	NA
Rate*of SUID	1.7 (1.6, 1.8)	0.36 (0.28, 0.47)	1.4 (1.36, 1.53)	1.24 (0.59, 1.52)

*Rates per 1,000 infant-years with exact 95% Poisson confidence intervals.

The table presents rates of apnea or SUID by two separate disease condition (bronchiolitis or apnea) comparisons that are not mutually exclusive groups.

### Temporal relationships

**Fig 1:**
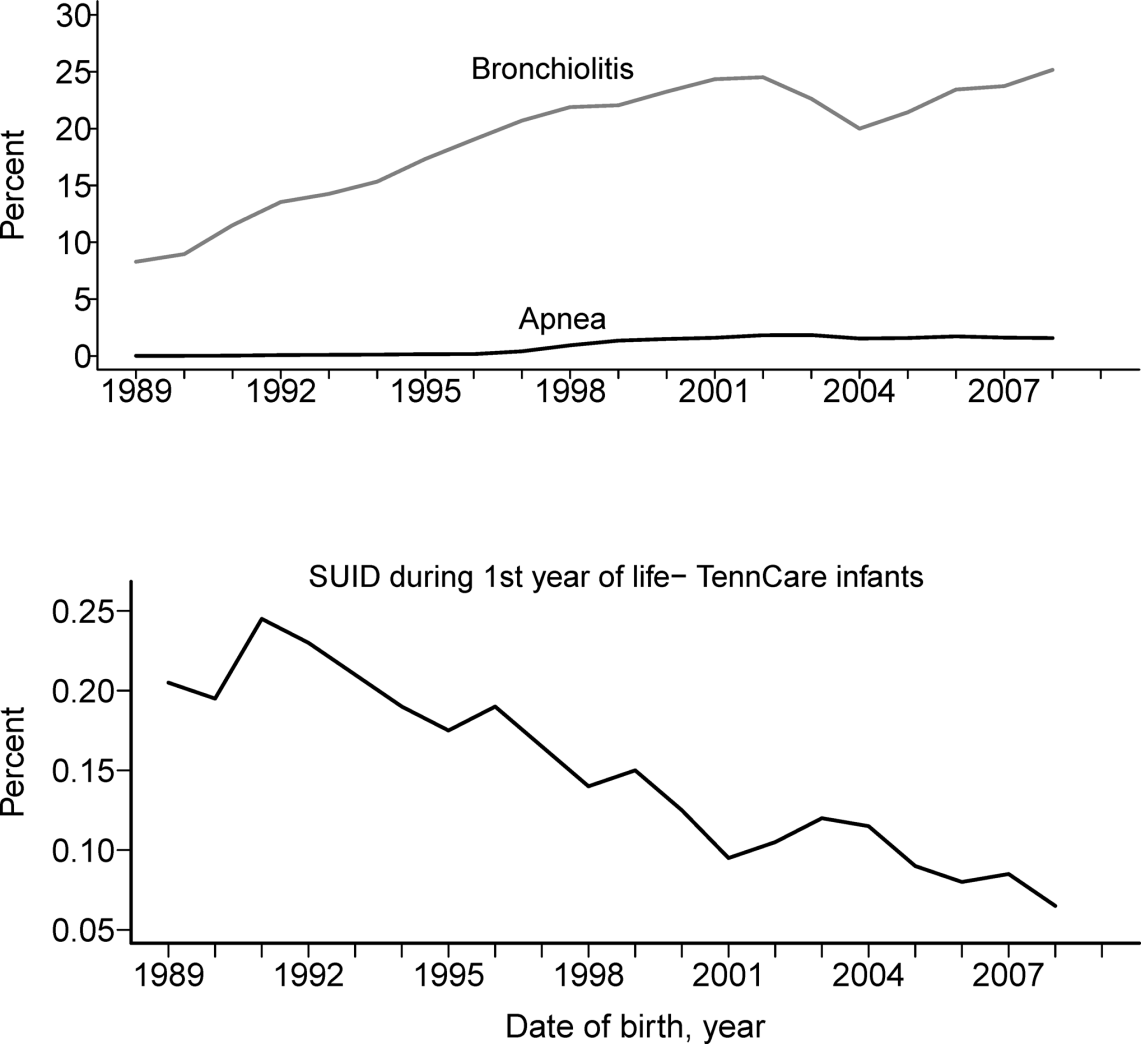
Percent of infants in the cohort with bronchiolitis, apnea and SUID events by birth years, 1989–2009. **a.** Percent of infants in the cohort with bronchiolitis and apnea events increased over time. **b.** The percent of infants in the cohort who died of SUID decreased over time. A moving average smoothing technique was applied to help visualize long-term trends.

Apnea was more likely to occur temporally near or in conjunction with bronchiolitis (S1 Fig). The percent of infants in the cohort over time with each exposure and SUID are shown in Fig 1 (a and b). Over the 20 year period, the percent of cases of apnea and bronchiolitis continually increased, while the percent of SUID cases dropped substantially (S2 Fig).

### Event patterns

**Fig 2:**
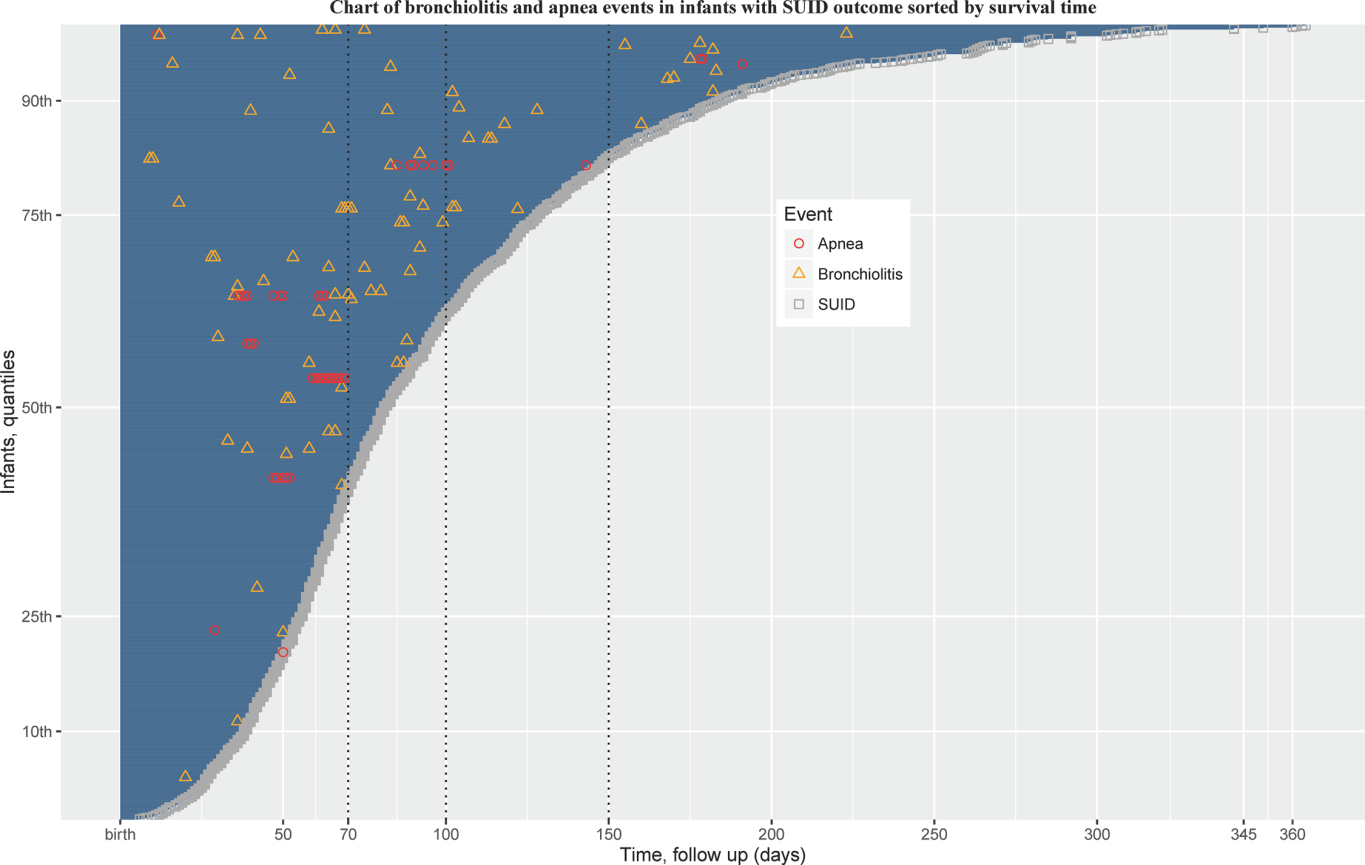
Events chart of the timing of bronchiolitis^a^ and apnea visits for all SUID cases (N = 1,195). Events are ordered by time (days) from birth to death. The y-axis shows quantiles of infants by time to death from SUID. Red circles represent an apnea event, yellow triangles represent a bronchiolitis event, and gray squares represent death from SUID forming the end of the time course. ^a^Multiple bronchiolitis health care encounters for an infant within a 7-day period were considered as a single bronchiolitis event. The time gap between bronchiolitis and SUID can be gauged from the x-axis of this figure.

We examined patterns of apnea and bronchiolitis healthcare visits in all 1,195 infants with SUID (Fig 2). Greater than 60% of apnea or bronchiolitis events occurred among infants with at least 70 days of survival time. Of the 1,195 infants with SUID, 59 had at least one bronchiolitis event and 17 had more than one bronchiolitis event. There were 4 infants with SUID who had at least one bronchiolitis event and at least one apnea event outside of their birth hospitalization. In SUID infants, when comparing those with bronchiolitis (n = 59) vs. those without bronchiolitis (n = 1136), a higher percentage of infants with SUID with bronchiolitis had mothers with asthma (maternal asthma available in subset, N = 7/33, 21%) compared to those who died of SUID but did not have a bronchiolitis event (N = 43/593, 7%) (p = 0.004). On the other hand, among infants who died of SUID with precedent bronchiolitis (n = 59) compared with those without precedent bronchiolitis (n = 1,136), maternal smoking (44% vs. 48%, p = 0.5), gestational age (266, [IQR: 253, 280] vs.274 [260, 280], p = 0.2), and birth weight (2722 gm [2344, 3331] vs 2977 gm [2466, 3318] p = 0.2) were not significantly different.

### Impact of second-hand smoke exposure and maternal asthma

The adjusted CPH model used individual data rather than ecological data, and was able to account for variation in follow-up time resulting from SUID occurring at different ages. In infants with no bronchiolitis visits, maternal smoking vs no maternal smoking was significantly associated with increased risk of SUID [aHR 2.38 (95% CI: 2.11, 2.67, p-value <0.001)]. Infants with at least 1 bronchiolitis visit and a mother who smoked, when compared to those with mothers who did not smoke did not have a significant association with increased SUID [aHR 1.51 (95% CI: 0.90, 2.53, p-value = 0.12)]. The interaction term for bronchiolitis and maternal smoking was marginally significant, p-value of 0.092.

Infants with no bronchiolitis visits and who had a mother who did not have asthma had no increased risk of SUID [aHR 1.11 (95% CI:0.82, 1.52, p-value = 0.50)]. The interaction term for bronchiolitis visits and maternal asthma was also marginally significant, p-value of 0.09.

The results of this model are given in Table 3. In infants with at least one bronchiolitis visit, having a mother with asthma was associated with higher risk of SUID [hazard ratios for those with mothers who did not have asthma or did not smoke, *asthma*: HR = 2.40, 95% CI: 1.04, 5.54, p-value = 0.039) whereas maternal smoking was associated with higher risk of SUID, *smoking*: HR = 2.38 (2.11, 2.67, p-value <0.001) among those without any bronchiolitis].

**Table 3:** Association of maternal smoking or maternal asthma with SUID outcome by infant bronchiolitis status, Cox Proportional Hazards model results. ^a^

	≥ 1 bronchiolitis visit		No bronchiolitis visit	
	HR (95%CI)	P value	HR (95%CI)	P value
Maternal smoking vs No* smoking	1.51 (0.90, 2.53)	0.12	2.38 (2.11, 2.67)	<0.001
Maternal asthma vs No asthma **†	2.40 (1.04, 5.54)	0.040	1.11 (0.82, 1.52)	0.50

^a^Separate Cox Proportional Hazards regression models were conducted for maternal smoking and bronchiolitis and maternal asthma († for maternal asthma subset with data) and bronchiolitis interaction tests (P values for interactions 0.092* and 0.090**); covariates included for adjustments were infant sex, gestational age, birth weight, year of birth.

## Discussion

This analysis represents the largest retrospective study of temporal trends in bronchiolitis, apnea and SUID to date. We used ecological methods and statistical modeling to investigate the temporal relationships between these events among infants in a large population-based birth cohort covering 20 years. While both SUID and bronchiolitis occurred at higher rates in winter (when RSV circulates) than in summer, SUID did not have the pronounced epidemic peak in January that is seen with bronchiolitis events. Rates of bronchiolitis and apnea have increased over the last 20 years in this population, while rates of SUID have decreased. There was not a temporal relationship between either bronchiolitis or apnea, and SUID events. There was a strong temporal relationship, however, between bronchiolitis and apnea. Bronchiolitis visits occur more often near apnea events in this population, supporting the previous findings that bronchiolitis may be one factor in triggering central apnea in infants [7]. It is important to note that many infants who died from SUID were premature, and factors associated with development in addition to the environmentmay influence risk.

There were limitations inherent in studying rare events such as SUID using healthcare data, in that subjects may have had other underlying conditions associated with the outcome. While this study supports the framework of an association between bronchiolitis and apnea, we did not see a strong ecological relationship between these events and SUID. These results, however, do not exclude a relationship between RSV, apnea and SUID, we just did not demonstrate one in this study. While an ecological study design and analysis are useful for discerning directionality, general, and temporal trends over time, further individual-level studies are needed. For example, an individual-level study with a prospective cohort design, while difficult because of the rarity of these events, would allow for the collection of specimens for biomarker analysis, and measures of breathing and apnea and thus analyses that could address etiological or causal relationships. Moreover, we could not confirm the viral agent causing bronchiolitis, and milder infections not easily identified using claims data could also likely contribute to apnea and/or SUID. Previous research hypothesized that in the first few months of life young infants can have apnea due to viral infections in the absence of visible respiratory distress [20].

The potential for an effect modification as observed by the increased risk of SUID among those infants with bronchiolitis who have mothers with asthma suggests that genetic predisposition may increase risk for SUID. A better understanding of both SUID and the neurologic sequelae of RSV or other viruses will likely be important in furthering our understanding of these uncommon, but highly morbid early life events.

## Supporting Information

S1 Fig Temporal relationships. Only infants with both bronchiolitis and apnea events are included. (A) Temporal relationship of infant apnea healthcare events to first infant bronchiolitis event. The infant’s first bronchiolitis visit is marked as day zero, subsequent bronchiolitis events are not included. Bars represent frequency of clinical indication of apnea occurring within N days of the first bronchiolitis visit. The x-axis represents the time in days between a bronchiolitis health care visit and an apnea healthcare visit. The bar at zero represents apnea visits that occurred on the same day as their first bronchiolitis visit. (B) The same data and analysis method as in A, with apnea as the index visit. Bars represent the frequency of bronchiolitis events before and after the first apnea healthcare visit.
(PDF)

S2 Fig Cases by month. The above graph shows all SUID, apnea, and bronchiolitis health care visits from 1989–2009 collapsed by month of year to investigate seasonal patterns. The y-axis shows SUID and bronchiolitis cases per 100 total SUID or bronchiolitis cases (not a population-based rate).
(PDF)

S1 Table Exposure variables. Percent of exposure variables (bronchiolitis and apnea healthcare visits) among premature and term infants. Prematurity is defined as being born at less than 37 weeks gestation.
(DOCX)
