# Supplementary material for: Seasonal Timing of Infant Bronchiolitis, Apnea and Sudden Unexplained Infant Death
Source: PLoS One. 2016 Jul 12;11(7):e0158521. doi: 10.1371/journal.pone.0158521 (PMC4942135; doi:10.1371/journal.pone.0158521)
Supplement: S1 Fig — Only infants with both bronchiolitis and apnea events are included. (A) Temporal relationship of infant apnea healthcare events to first infant bronchiolitis event. The infant’s first bronchiolitis visit is marked as day zero, subsequent bronchiolitis events are not included. Bars represent frequency of clinical indication of apnea occurring within N days of the first bronchiolitis visit. The x-axis represents the time in days between a bronchiolitis health care visit and an apnea healthcare visit. The bar at zero represents apnea visits that occurred on the same day as their first bronchiolitis visit. (B) The same data and analysis method as in A, with apnea as the index visit. Bars represent the frequency of bronchiolitis events before and after the first apnea healthcare visit. (PDF) [file pone.0158521.s001.pdf]

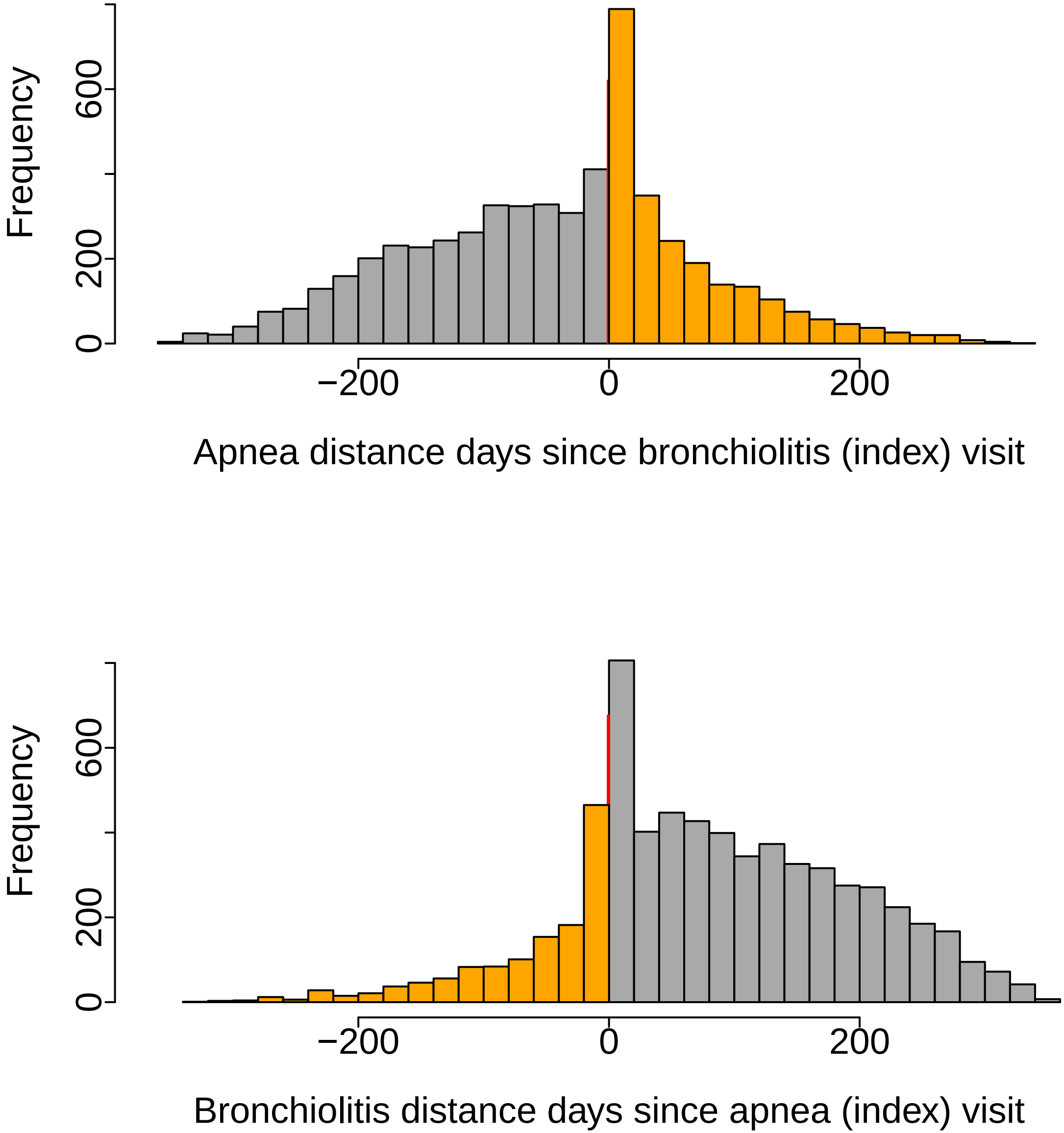
Frequency
0
200
600
−200
0
200
Apnea distance days since bronchiolitis (index) visit
Frequency
0
200
600
−200
0
200
Bronchiolitis distance days since apnea (index) visit
