# Supplementary figures and images for: Seasonal Timing of Infant Bronchiolitis, Apnea and Sudden Unexplained Infant Death
Source: PLoS One. 2016 Jul 12;11(7):e0158521. doi: 10.1371/journal.pone.0158521 (PMC4942135; doi:10.1371/journal.pone.0158521)

Years 1989–2010

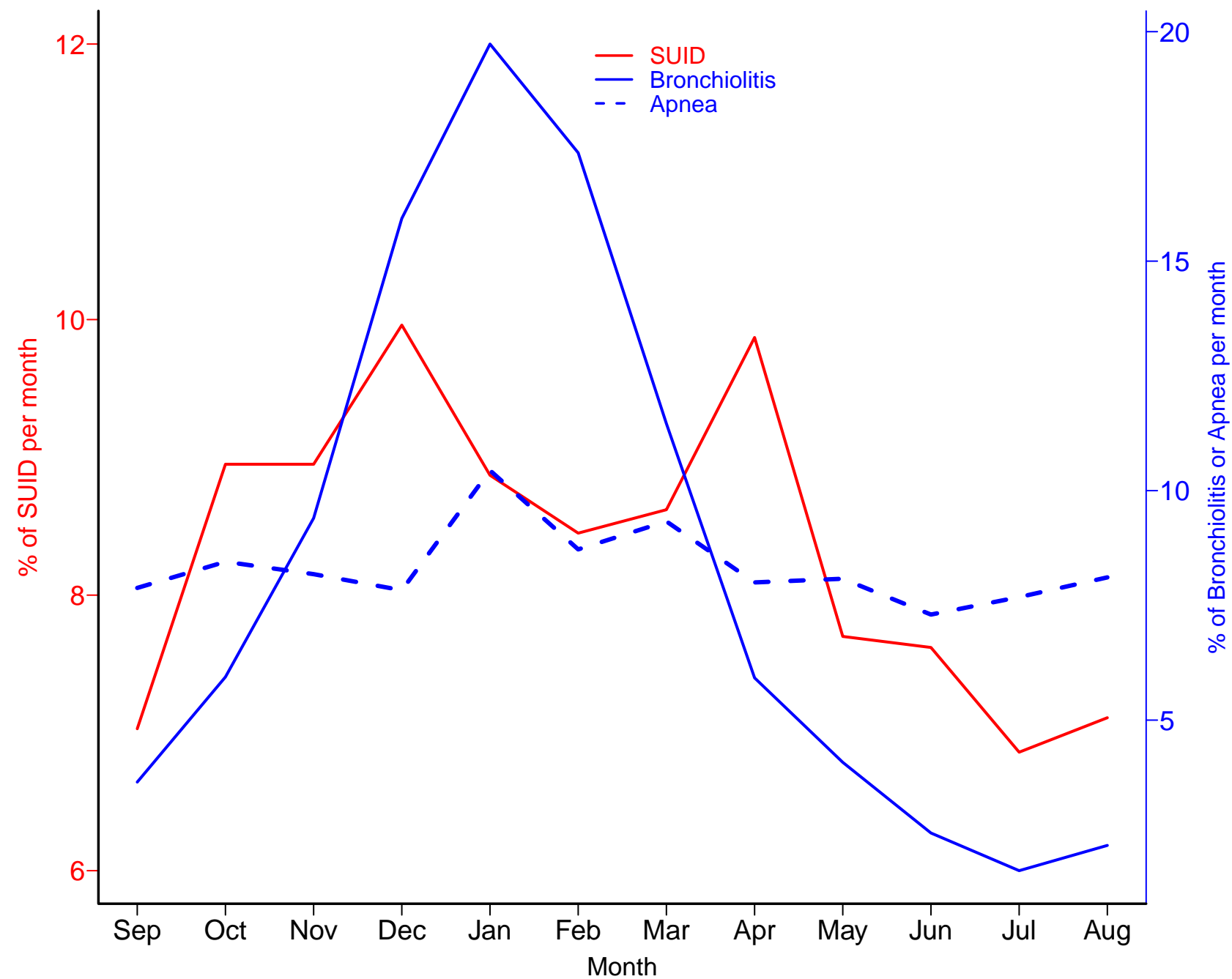

Supplement: S2 Fig — The above graph shows all SUID, apnea, and bronchiolitis health care visits from 1989–2009 collapsed by month of year to investigate seasonal patterns. The y-axis shows SUID and bronchiolitis cases per 100 total SUID or bronchiolitis cases (not a population-based rate). (PDF) [file pone.0158521.s002.pdf]
