# Supplementary material for: Seasonal Timing of Infant Bronchiolitis, Apnea and Sudden Unexplained Infant Death
Source: PLoS One. 2016 Jul 12;11(7):e0158521. doi: 10.1371/journal.pone.0158521 (PMC4942135; doi:10.1371/journal.pone.0158521)
Supplement: S1 Table — Percent of exposure variables (bronchiolitis and apnea healthcare visits) among premature and term infants. Prematurity is defined as being born at less than 37 weeks gestation. (DOCX) [file pone.0158521.s003.docx]

**SUPPLEMENTARY DATA**

**ICD-9 codes used to define infant status:** Infants were defined as having a bronchiolitis visit in the first year of life based on an ICD-9 code of 466.1 and/or 480.1 as one of the top three codes for an outpatient visit, emergency department visit, or hospitalization. Apnea visits were assigned based on the following codes: 327.2/372.0 (organic sleep apnea), 786.03 (apnea), 770.81 (primary apnea of newborn), 770.82 (other apnea of newborn), 327.29 (other organic sleep apnea), 780.57 (unspecified sleep apnea), 327.21 (primary central sleep apnea), 780.51 (insomnia with sleep apnea, unspecified), and/or 780.53 (hypersomnia with sleep apnea, unspecified) during any visit type. SUID was determined by a code of 798 for any visit type.

**Table S1:** Percent of exposure variables (bronchiolitis and apnea healthcare visits) among premature and term infants.

|  | **Premature** (N= 114,577) | **Term** (N=720,018) |
| --- | --- | --- |
| **Any bronchiolitis visit** | 27,217 (24%) | 135,390 (19%) |
| **Bronchiolitis hospitalization*** | 8,080 (7%) | 31,355 (4%) |
| **Any apnea visit** | 3,526 (3%) | 4,613 (1%) |
| **SUID** | 299 (0.3%) | 896 (0.12%) |

*Infants who were admitted to the hospital, not those who had a hospital visit lasting 23 hours or less.

Prematurity is defined as being born at less than 37 weeks gestation.
